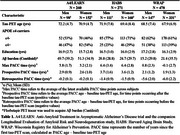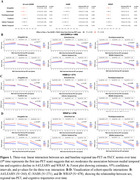# Sex moderates the association between medial temporal tau pathology and cognitive decline: A multi‐cohort study

**DOI:** 10.1002/alz70856_100370

**Published:** 2025-12-24

**Authors:** Annie Li, Hannah M Klinger, Mabel Seto, Colin Birkenbihl, Michael J. Properzi, Michelle E. Farrell, Aaron P. Schultz, Emma G Thibault, Robert A. Rissman, Diana L Townsend, Kathryn V Papp, Rebecca E. Amariglio, Hyun‐Sik Yang, Rema Raman, Oliver Langford, Michael C. Donohue, Tobey J. Betthauser, Rebecca E. Langhough, Erin M. Jonaitis, Karly Alex Cody, Sterling C Johnson, Dorene M. Rentz, Keith A. Johnson, Reisa A. Sperling, Rachel F. Buckley, Gillian T Coughlan

**Affiliations:** ^1^ Massachusetts General Hospital, Harvard Medical School, Boston, MA, USA; ^2^ Alzheimer's Therapeutic Research Institute, University of Southern California, San Diego, CA, USA; ^3^ Brigham and Women's Hospital, Boston, MA, USA; ^4^ University of Wisconsin‐Madison School of Medicine and Public Health, Madison, WI, USA; ^5^ Melbourne School of Psychological Sciences, University of Melbourne, Melbourne, VIC, Australia

## Abstract

**Background:**

Existing positron emission tomography(PET) studies suggest that women exhibit greater regional tau burden than men, but the implications of this sex difference on cognitive trajectories remain elusive. We investigated sex differences on cognitive trajectories as a function of tau burden.

**Method:**

Participants were 1009 clinically normal adults(mean age: 69.5; 363 *APOE*ε4 carriers; Table 1) from the Anti‐Amyloid Treatment in Asymptomatic Alzheimer's Disease trial, the companion Longitudinal Evaluation of Amyloid Risk and Neurodegeneration study(A4/LEARN), the Harvard Aging Brain Study(HABS) and the Wisconsin Registry for Alzheimer's Prevention(WRAP), with at least one baseline tau‐PET scan and one or more cognitive visits (mean (SD) = 3.2(3.9) years, range=‐9.9‐11.1 years). Tau‐PET imaging utilized Flortaucipir(FTP) in A4/LEARN and HABS, while MK‐6240 was used in WRAP. Six Alzheimer's disease‐related tau regions were selected for analysis: Entorhinal cortex, amygdala, parahippocampal gyrus, fusiform gyrus, inferior temporal and middle temporal gyri. Cognitive outcomes were measured using Preclinical Alzheimer's Cognitive Composite(PACC) scores. Random‐effects regression models estimated the interaction between sex(male/female), regional tau(continuous), and PACC time(=PACC age – baseline tau‐PET age) adjusting for baseline tau‐PET age and education over time, including participant‐specific intercepts and slopes.

**Result:**

The three‐way effects are summarized in the forest plot(Figure 1A) and visualized in model‐predicted trajectories(Figure 1B, 1D for A4/LEARN and WRAP; Figure 1C for HABS). In A4/LEARN and WRAP**,** women with higher baseline amygdala and parahippocampal tau exhibited significantly faster cognitive decline compared to men, with additional effects observed in the entorhinal cortex for A4/LEARN. At low tau levels, women maintained higher PACC performance relative to men. In HABS, sex did not moderate the association between baseline tau and cognitive decline. Covarying for *APOE*ε4‐status and Aβ burden(Centiloid) did not attenuate existing sex effects.

**Conclusion:**

Among those with higher medial temporal tau, women exhibit faster cognitive decline compared to men. These findings suggest that early intervention with anti‐tau therapies may be particularly important for women who exhibit medial temporal tauopathy. Future research should investigate sex differences in the spread of medial temporal tau to the neocortex, which may further elucidate faster cognitive decline among women.